# The Prevalence of and Factors Associated With Neck Pain Among Jazan Adult Population

**DOI:** 10.7759/cureus.28008

**Published:** 2022-08-14

**Authors:** Zenat Khired

**Affiliations:** 1 Surgery, Jazan University, Jazan, SAU

**Keywords:** preva, risk factors, prevalence, musculoskeletal, neck pain

## Abstract

Background

Neck pain is considered one of the main musculoskeletal conditions affecting the population worldwide. However, it is difficult to identify the precise causes of pain.

Objective

This study aimed to estimate the prevalence of neck pain and its associated factors in the adult population and surrounding districts.

Methods

This cross-sectional study was conducted in Jazan city and the surrounding districts of the Kingdom of Saudi Arabia. A total of 443 adults of both sexes participated in an online questionnaire designed to determine the prevalence of neck pain and the significance of the relationship between neck pain and specific risk factors. The collected data included the participants' characteristics (personal, socioeconomic, demographic, and work-related aspects).

Results

Neck pain was highly prevalent among adults in Jazan city and the surrounding districts, as 347 of 443 participants (78.3%) experienced neck pain, while 96 (21.7%) did not suffer from neck pain. There was a significant relationship between the most common physical positions while using electronic devices and reading and the prevalence of neck pain (p = 0.015). The most common position accompanied by neck pain was the sitting position compared to the positions of lying, walking, or standing (79.7% vs. 67.9%, 60%, and 0%, respectively). No significant association was observed between the prevalence of neck pain and sex, age, monthly income, place of residence, smoking, or the number of hours spent on electronic devices or reading.

Conclusion

This study revealed a high prevalence of neck pain among adults in Jazan, Saudi Arabia, and a remarkable association with people who spend many hours daily using electronic devices, reading, performing work, sitting for a long time, and with lack of exercise.

## Introduction

Neck pain is a common musculoskeletal problem with a lifetime prevalence of 14-70% in the general population [1,2]. The Global Burden of Disease report accounts for the fourth highest number of years lived with a disability [3]. The direct and indirect economic costs of neck pain have encouraged researchers to study its prevalence and risk factors associated with neck pain in the general population. Although acute episodes of neck pain may resolve completely, recurrence and chronicity occur frequently. Poor recovery from acute neck pain is more common than previously thought [4]. The risk factors for neck pain typically occur insidiously. They are usually multifactorial, involving one or more of the following: ergonomic (excessive physical activity, use of force and vibration, poor posture, repeated movement), personal (age, BMI, genome, history of musculoskeletal pain), behavioral (smoking and physical activity level), and psychosocial (job satisfaction, stress level, anxiety, and depression). Moreover, for most students, neck pain can be greater in those who lead sedentary lives [2]. For example, in a study conducted among Ethiopian school teachers, the teacher with static head-down posture, elevated arm over the shoulder, and hypertension were associated with significant shoulder and neck pain [5]. Many studies indicate a higher incidence of neck pain among women. The older the female, the higher the degenerative changes and the neck pain [6]. The different results of observational studies could be due to varying definitions, such as the region and duration of neck pain. It may also be caused by methodological differences, such as non-comparable population samples, differing response rates, and the overall quality of the studies. It could also cause bias and explain the discrepancies [7,8]. Pain is either acute or chronic, depending on the primary cause. If it is chronic, its duration lasts for more than three months. Feeling pain in a particular position can be mild or sharp. Some cases can vanish from days to weeks, and some may last longer, leading to complications.

The pain is always at the starting point in the neck and can radiate to nearby areas, such as the chest, shoulders, and head. Therefore, pain can be accompanied by other symptoms [9,10]. Therefore, this study aimed to estimate the prevalence of neck pain among the adult Jazan population and its associated factors to create available background information about neck pain prevalence and risk factors among the Jazan city population.

## Materials and methods

Study design and population characteristics 

This cross-sectional study was conducted between July and August 2020. It was carried out in Jazan city and the surrounding districts of Saudi Arabia, with a population of 1,567,547 in the 2017 census. The study targeted a population aged 15 years and older, using an online web-based survey addressing different social media (WhatsApp, Twitter, Facebook, Instagram, and Snapchat) distributed in the Jazan region. The data was collected after getting the IRB approval from the Standing Committee for Scientific Research Ethics, Jazan University (HAPO-10-Z-001) (reference number REC42/1/016, 2020).

Sampling and data collection 

The sample size was calculated as 400 individuals using the sample size formula for cross-sectional studies. The study used the parameters of p= 50% to compute the maximum sample size. A refusal rate of 20% was assumed in this study based on the sample size formula for a cross-sectional study design. The margin of error selected was 0.05, with a 95% CI. An online web-based survey (snowball type) in simple Arabic language was designed to determine the prevalence and associated risk factors of neck pain. The questionnaires included demographic questions such as sex, age, monthly income, place of residence, and smoking history. Questions related to neck pain and associated factors such as how many hours they spend using electronic devices, reading, and the most common position during its usage were included. Duration of neck pain, use of painkillers, visiting doctors due to pain, associated symptoms such as headache, the relationship between neck pain and psychological stress and ability to concentrate, questions related to trauma, lifting heavy objects, long-distance driving, and questions related to practicing exercise such as walking, running, and swimming were also included.

Statistical analysis 

Data were collected electronically through an online Google form questionnaire and distributed through the social media database. Data were analyzed by using the SPSS package v19. Frequencies and percentages were used to analyze the selected sociodemographic data. Differences in demographic and the study variables were analyzed using a t-test. In addition, a Chi-squared test was used to determine the significance of the relationship between neck pain and specific risk factors. A p-value equal to or less than 0.05 is considered statistically significant.

## Results

A total of 443 adults (15 years and older) in Jazan city and the surrounding districts were included in the study. Eighty-nine (20.1%) participants were male, and 354 (79.9%) were female. One hundred seventy-nine of them (39.5%) were aged between 15 and 30 years, 246 (55.5%) were aged between 31 and 50 years, and 22 (5%) were aged 51 years or older. Regarding monthly income, 43 (9.7%) of the participants had an income less than 2000 SR, 78 (17.6%) had an income between 2000 and 5000 SR, 136 (30.7%) had an income between 5000 and 10000 SR, and 186 (42%) had an income of more than 10000 SR. Regarding the place of residency, the largest number of participants were from Abu Ariesh, 139 (31.4%); Jazan city, 74 (16.7%); and Samtha, 57 (12.9%). Thirty-seven (8.4%) participants were smokers, and 406 (91.6%) were non-smokers. Table 1 shows the sociodemographic profiles of the participants.

**Table 1:** Sociodemographic profile of the participants (n = 443).

Socio-demographic characteristics		n	%
Gender			
Male		89	20.1
Female		354	79.9
Age			
15-30 years		175	39.5
31-50 years		246	55.5
51 years or older		22	5
Monthly Income			
Less than 2000 SR		43	9.7
Between 2000-5000 SR		78	17.6
Between 5000-10000 SR		136	30.7
More than 10000 SR		186	42
Place of residency			
Jazan		74	16.7
Sabia		24	5.4
Abu Ariesh		139	31.4
Al Darb		37	8.4
Beish		9	2
Samtah		57	12.9
Ouhd Al-Msaraha		23	5.2
Others		80	18.1
Smoking			
Yes		37	8.4
No		406	91.6

**Figure 1:**
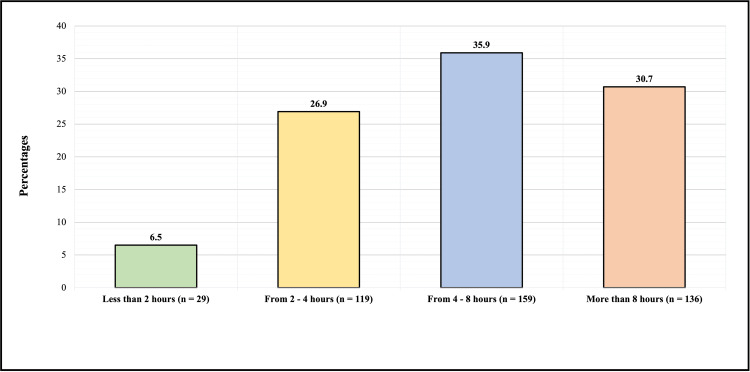
Daily hours spent by the participants on electronic devices and reading.

Figure 1 shows the number of hours the participants spend on electronic devices and reading daily. Twenty-nine (6.5%) participants spent less than two hours on electronic devices/reading, 119 (26.9%) spent 2-4 hours, 159 (35.9%) spent 4-8 hours, and 136 (30.7%) spent more than 8 hours.

**Figure 2:**
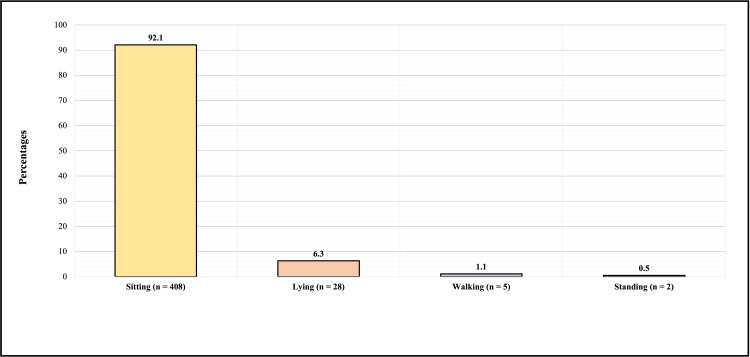
Participants most common positions while reading and using electronic devices.

Figure 2 shows the participants' most common positions while using electronic devices/reading. Four hundred and eight (92.1%) reported that sitting was the most common position during electronic devices/reading, 28 (6.3%) reported lying down, 5 (1.1%) reported walking, and 2 (0.5%) reported standing as the most common position while using electronic devices/reading.

**Figure 3:**
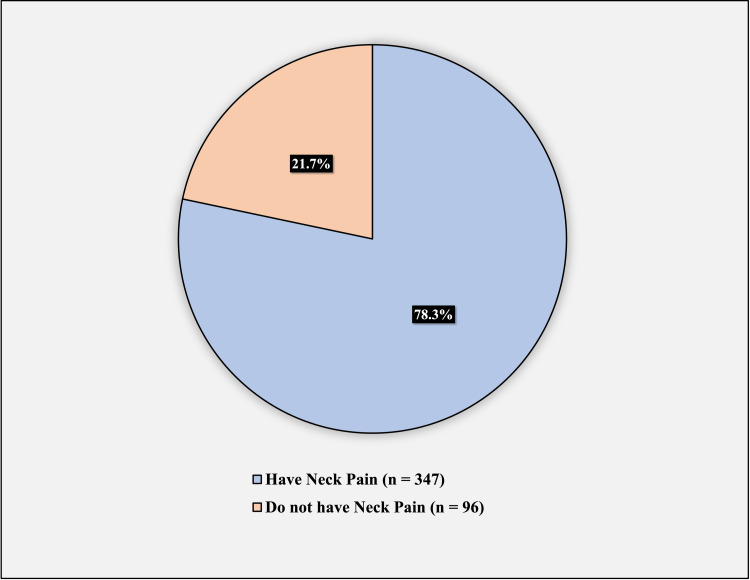
Prevalence of neck pain.

Figure 3 illustrates the prevalence of neck pain; 347 participants (78.3%) had neck pain, and 96 (21.7%) did not have neck pain.

Table 2 shows the neck pain-related history of the participants with neck pain. When asked about the duration of neck pain, 198 (57.1%) reported hours, 114 (32.9%) reported days, 16 (4.6%) reported a week, and 19 (5.5%) reported that the pain was chronic and daily. Regarding the assessment of the severity of the pain, 80 (23.1%) stated it was mild, 210 (60.5%) stated it was moderate, 53 (15.3%) stated it was severe, and 4 (1.2%) stated it was very severe. Forty-two participants (40.9%) stated that they used painkillers, while 20.5 (59.1%) reported not using painkillers. One hundred twenty-one (85.2%) of those who used painkillers said it relieved their pain, and 21 (14.8%) stated they did not. Eighty-nine participants (25.6%) stated that they visited a doctor once for their neck pain, while 258 (74.4%) reported that they did not.

**Table 2:** Neck pain-related history (n = 347).

Question	n	%
Q1/ How long does your neck pain usually lasts?		
Hours	198	57.1
Days	114	32.9
A week	16	4.6
Chronic on daily basis	19	5.5
Q2/ How would you rate the severity of your neck pain?		
Mild	80	23.1
Moderate	210	60.5
Severe	53	15.3
Very severe	4	1.2
Q3/ Do you use pain killers for your neck pain?		
Yes	142	40.9
No	205	59.1
Q4/ If you use pain killers, does it relief your neck pain? (n = 142)		
Yes	121	85.2
No	21	14.8
Q5/ Have you ever visited a doctor for your neck pain?		
Yes	89	25.6
No	258	74.4
Q6/ Which of the following do you think relates to neck pain?		
Inappropriate posture	197	56.8
Using electronic devices	114	32.9
Diseases	32	9.2
Previous trauma accident	2	0.6
All of the previous	2	0.6
Q7/ Do you suffer from headache alongside with your neck pain?		
Yes	237	68.3
No	110	31.7
Q8/ Have you ever tried changing your position of using electronic devices/reading to relieve your neck pain?		
Yes	275	79.3
No	72	20.7
Q9/ Is there a relationship between neck pain and your ability to concentrate?		
Yes	240	69.2
No	107	30.8
Q10/ Do you think there is a relationship between neck pain and psychological stress?		
Yes	222	64
No	125	36

When the participants were asked about what could be related to neck pain, 197 (56.8%) thought inappropriate posture, 114 (32.9%) thought of using electronic devices, 32 (9.2%) thought of disease, 2 (0.6%) thought of previous trauma/accident, 2 (0.6%) thought that all previous could be related. Two hundred thirty-seven patients (68.3%) reported headaches along with neck pain. In contrast, 275 (79.3%) participants reported that they tried to change their posture while using electronic devices/readings to relieve neck pain. Of the total number of participants, 240 (69.2%) reported a relationship between neck pain and the ability to concentrate, while 222 (64%) thought there was a relationship between neck pain and psychological stress.

Table 3 displays the trauma history and behaviors related to neck pain among the participants with neck pain. Thirty-two (9.2%) of the participants reported having a history of neck trauma, while 99 (28.5%) reported lifting heavy objects, and 179 (51.6%) said they sometimes did. Twenty-seven (7.8%) reported driving long times daily, and 46 (13.3%) reported that they sometimes do. One hundred and fifteen (33.1%) participants reported having trouble sleeping at night because of neck pain, while 142 (40.9%) reported that they sometimes do. When asked about working out, 98 (28.2%) reported working out for half an hour, 58 (16.7%) reported working out for an hour, 10 (2.9%) reported working out for two hours, two (0.6%) reported working for three hours, one (0.3%) reported working out for more than three hours, and 178 (51.3%) reported not working out. Sixteen participants (4.6%) reported that their neck pain was related to working out, 261 (75.2%) reported that it was not, and 70 (20.2%) reported that it was sometimes.

**Table 3:** Trauma history and behaviors related to neck pain among participants with neck pain (n = 347).

Question	n	%
Q1/ Have you ever had accident/trauma to your neck?		
Yes	32	9.2
No	315	90.8
Q2/ Do you lift any heavy objects?		
Yes	99	28.5
No	69	19.9
Sometimes	179	51.6
Q3/ Do you drive for long hours on daily basis?		
Yes	27	7.8
No	274	79
Sometimes	46	13.3
Q4/ Do you have trouble sleeping because of your neck pain?		
Yes	115	33.1
No	90	25.9
Sometimes	142	40.9
Q5/ How long do you spend in exercising?		
Half an hour	98	28.2
An hour	58	16.7
2 hours	10	2.9
3 hours	2	0.6
More than 3 hours	1	0.3
I don't work out	178	51.3
Q6/ Is your neck pain related to working out?		
Yes	16	4.6
No	261	75.2
Sometimes	70	20.2
Q7/ Do you have a job (work)?		
Yes	261	75.2
No	86	24.8
Q8/ Do you think your neck pain is related to your work? (n = 261)		
Yes	147	56.3
No	46	17.6
Maybe	68	26.1
Q9/ The nature of your work demands? (n = 261)		
Sitting for long hours	123	47.1
Sitting in an uncomfortable position	17	6.5
Standing up for long hours	32	12.3
Moving around a lot with little rest	37	14.2
Heavy labor work for long hours	3	1.1
Prefer not to answer	49	18.8
Q10/ Do you have any pshychiatric disorder related to your work (stress, anxiety)?		
No	93	35.6
Yes, I currently have	104	39.8
Yes, in the past (job-related)	17	6.5
Yes, in the past (not job-related)	17	6.5
I refrain from answering	30	11.5

Two hundred and sixty-one participants (75.2%) reported that they had a job, 147 (56.3%) thought their neck pain was related to their work, 68 (26.1%) thought it could be, and 46 (17.6%) thought it was not. As for the nature of the work, for those who had a job, 123 (47.1%) reported sitting for long hours, 17 (6.5%) reported sitting in an uncomfortable position, 32 (12.3%) reported standing up for long hours, 37 (14.2%) reported moving around a lot with little rest, 3 (1.1%) reported heavy labor work for long hours, and 49 (18.8%) preferred not to answer. When asked if they had work and are having any psychiatric disorder related to their work (such as anxiety and stress), 93 (35.6%) stated that they did not, 104 (39.8%) reported yes that they had at the time of answering the survey, 17 (6.5%) stated yes that they had in the past (job-related), 17 (6.5%) stated yes that they had in the past (not job-related), and 30 (11.5%) refrained from answering. 

**Figure 4:**
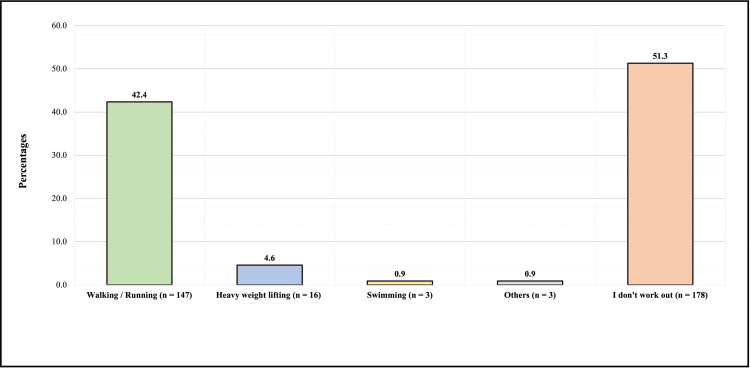
Type of exercise practiced by participants who experienced neck pain.

**Figure 5:**
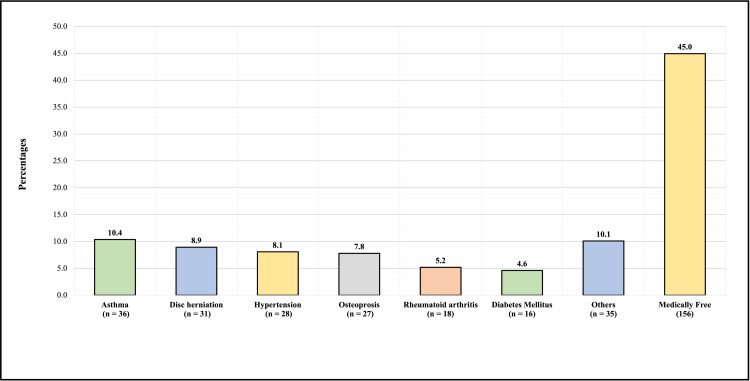
Medical history of participants who had neck pain.

Figure 4 shows the type of exercise practiced by participants who experienced neck pain. One hundred forty-seven (42.4%) reported walking/running, 16 (4.6%) reported heavy weightlifting, 3 (0.9%) reported swimming, and 3 (0.9%) reported other types of exercise, and 178 of them (51.3%) reported that they did not work out. Regarding the medical history of the participants with neck pain, 36 (10.4%) had asthma, 31 (8.9%) had disc herniation, 28 (8.1%) had hypertension, 27 (7.8%) had osteoporosis, 18 (5.2%) had rheumatoid arthritis, 16 (4.6%) had diabetes mellitus, 35 (10.1%) had other diseases, and 156 (45%) had no illness/disease (Figure 5).

Table 4 shows the association between the prevalence of neck pain and sociodemographic factors. There was a significant relationship between using electronic devices and reading and the prevalence of neck pain (p = 0.015). The most common position accompanied by neck pain was the sitting position compared with the position of lying, walking, or standing (79.7% vs. 67.9%, 60%, and 0%, respectively). No significant association was observed between the prevalence of neck pain and sex, age, monthly income, place of residence, smoking, or the number of hours spent on electronic devices or reading.

**Table 4:** The association between the prevalence of neck pain and sociodemographic factors.

Socio-demographic characteristics	Do you suffer from neck pain?		P-value
	Yes	No	
Gender			0.435
Male	67 (75.3%)	22 (24.7%)	
Female	280 (79.1%)	74 (20.9%)	
Age			0.085
15-30 years	129 (73.7%)	46 (26.3%)	
31-50 years	198 (80.5%)	48 (19.5%)	
51 years or older	20 (90.9%)	2 (9.1%)	
Monthly income			0.614
Less than 2000 SR	35 (81.4%)	8 (18.6%)	
Between 2000 and 5000 SR	60 (76.9%)	18 (23.1%)	
Between 5000 and 10000 SR	102 (75%)	34 (25%)	
More than 10000 SR	150 (80.6%)	36 (19.4%)	
Place of residency			0.23
Jazan	55 (74.3%)	19 (25.7%)	
Sabia	17 (70.8%)	7 (29.2%)	
Abu Ariesh	112 (80.6%)	27 (19.4%)	
Al Darb	33 (89.2%)	4 (10.8%)	
Beish	7 (77.8%)	2 (22.2%)	
Samtah	47 (82.5%)	10 (17.5%)	
Ouhd Al-Msaraha	20 (87%)	3 (13%)	
Others	56 (70%)	24 (30%)	
Smoking			0.682
Yes	28 (75.7%)	9 (24.3%)	
No	319 (78.6%)	87 (21.4%)	
How many hours do you spend on using electronic devices/reading?			0.064
Less than 2 hours	19 (65.5%)	10 (34.5%)	
From 2 to 4 hours	88 (73.9%)	31 (26.1%)	
From 4 to 8 hours	125 (78.6%)	34 (21.4%)	
More than 8 hours	115 (84.6%)	21 (15.4%)	
What is the most common position you stay in during using electronic devices/reading?			0.015*
Standing	0	2 (100%)	
Sitting	325 (79.7%)	83 (20.3%)	
Walking	3 (60%)	2 (40%)	
Lying	19 (67.9%)	9 (32.1%)	

## Discussion

Neck pain is a common musculoskeletal health problem among the general population and employees. The mean lifetime prevalence of neck pain is estimated to be approximately 50%, and the one-month prevalence is 25% [7]. The current study aimed to estimate the prevalence of neck pain among the adult population aged 15 years and older living in Jazan city and the surrounding districts and analyze its associated factors. This study included 443 participants, 280 women and 67 men with neck pain. The incidence of chronic neck pain was higher in women than in men, which is in line with an earlier study that reported that predictors varied by sex. Among women, workplace bullying, frequent sleep problems, and overweight or obesity, in addition to previous acute neck pain and chronic low back pain, independently predicted the development of chronic neck pain [10]. Furthermore, the present study showed that the most common age affected by neck pain was 31-51 years. This conclusion is consistent with past research that has demonstrated that adolescents with neck pain are more likely to have similar symptoms in adulthood and that life-long chronic neck pain may have its origins in childhood [11,12]. The study showed that sitting while using electronic devices and reading is a common position of neck pain, as 79.7% of the participants reported. The main reasons reported by the respondents in this study for neck pain are extended periods of sitting and a lack of exercise. On the other hand, there was a notable relationship between people's position during electronic devices and the prevalence of neck pain (92.1%). This study suggests that inappropriate posture and electronic devices are risk factors for neck pain. These findings are consistent with the epidemiological studies that reported that prolonged computer work with a high number of computer users is more frequently associated with upper extremity symptoms. However, little is known about the link between computer-use-related factors and the onset and persistence of neck pain [13].

In a recent study conducted among computer users working from home during the COVID-19 pandemic, most participants sat on a chair or bed without a desk and worked for long hours while in faulty posture. As a result, 42.9% of them had pain in the neck, and upper back, with up to 41.9%, 24.8%, and 3.1% had mild, moderate, and severe functional limitations due to neck pain [14]. A recent study conducted in Turkey and published in 2021 indicated that the upper back and neck have a higher prevalence of musculoskeletal pain among smartphone users, particularly with smartphone addiction [15]. Lifestyle, physical factors, psychological factors, social factors, and improper sitting have been identified as the risk factors associated with neck and shoulder pain in different studies among students. Different studies consider improper sitting, psychological factors, lifestyle, physical, and social factors as risk factors associated with neck and shoulder pain [16,17,18]. Another study found evidence of an association between neck pain and sitting, twisting, or bending of the trunk, high job demand, low job control, low co-worker support, high and low skill direction, and low job [19].

This study has confirmed that neck pain is a significant health problem among the adult population in Jazan city and its surrounding districts. The prevalence of neck pain among the respondents was high (78.3%). The Abu Arish area was the place of residence, with the highest number of participants (112) reported suffering from neck pain. The present study also found that most neck pains were nontraumatic. The participants mentioned different factors they thought to cause neck pain, such as stress, lifting heavy items, driving, working, and psychological disorders. This finding is consistent with a study reporting that similar to low back pain, most cases of neck pain are nonspecific; that is, no specific somatic pathology causing pain can be identified [20].

A range of risk factors for neck pain related to physical workload, psychosocial factors, and health-related behaviors have been reported in the literature, primarily based on cross-sectional or case-control studies. In their review, it was found that there are associations between neck pain, distress, and anxiety. In addition, it was found to include female sex, older age in men, high job demand, low support at work, being an ex-smoker, and a history of low back and neck injuries [10]. On the other hand, there is a study among Finnish adults where the prevalence is chronic. On the other hand, a study conducted in Finland found that the incidence of chronic neck syndrome was related to a history of back, neck, or shoulder injury, as well as mental and physical stress at work [21]. The current study has several limitations. First, the prevalence estimate was specific to individuals complaining of chronic neck pain with no functional impairment. The study also did not include all districts surrounding Jazan city. However, the cross-sectional nature of data collection also precludes any conclusions about causality.

## Conclusions

Neck pain is prevalent among people living in Jazan city and surrounding districts. This study confirmed that neck problem is a significant health problem among the adult population in the Jazan area, with a range of risk factors such as lifestyle, improper sitting, and other factors. Further studies are needed to define the reasons for neck pain and how to treat it.
